# Notch1 Signaling Regulates the Proliferation and Self-Renewal of Human Dental Follicle Cells by Modulating the G1/S Phase Transition and Telomerase Activity

**DOI:** 10.1371/journal.pone.0069967

**Published:** 2013-07-29

**Authors:** Xuepeng Chen, Tianhou Zhang, Jiejun Shi, Ping Xu, Zexu Gu, Andrew Sandham, Lei Yang, Qingsong Ye

**Affiliations:** 1 Department of Orthodontics, Hospital of Stomatology, Zhejiang University, Hangzhou, Zhejiang, China; 2 Department of Stomatology, Second Affiliated Hospital of Medical College, Zhejiang University, Hangzhou, Zhejiang, China; 3 Institute of Zoology, Chinese Academy of Sciences, Beijing, China; 4 Department of Orthodontics, Qindu Stomatological College, Fourth Military Medical University, Xi’an, Shaanxi, China; 5 Department of Orthodontics, School of Medicine and Dentistry, James Cook University, Cairns, Queensland, Australia; National Cancer Institute, United States of America

## Abstract

Multipotent human dental follicle cells (HDFCs) have been intensively studied in periodontal regeneration research, yet the role of Notch1 in HDFCs has not been fully understood. The aim of the current study is to explore the role of Notch1 signaling in HDFCs self-renewal and proliferation. HDFCs were obtained from the extracted wisdom teeth from adolescent patients. Regulation of Notch1 signaling in the HDFCs was achieved by overexpressing the exogenous intracellular domain of Notch1 (ICN1) or silencing Notch1 by shRNA. The regulatory effects of Notch1 on HDFC proliferation, cell cycle distribution and the expression of cell cycle regulators were investigated through various molecular technologies, including plasmid construction, retrovirus preparation and infection, qRT-PCR, western blot, RBP-Jk luciferase reporter and cell proliferation assay. Our data clearly show that constitutively activation of Notch1 stimulates the HDFCs proliferation while inhibition of the Notch1 suppresses their proliferation *in vitro*. In addition, the HDFCs proliferation is associated with the increased expression of cell cycle regulators, e.g. cyclin D1, cyclin D2, cyclin D3, cyclin E1, CDK2, CDK4, CDK6, and SKP2 and the decreased expression of p27^ kip1^. Moreover, our data show that the G1/S phase transition (indicating proliferation) and telomerase activity (indicating self-renewal) can be enhanced by overexpression of ICN1 but halted by inhibition of Notch1. Together, the current study provides evidence for the first time that Notch1 signaling regulates the proliferation and self-renewal capacity of HDFCs through modulation of the G1/S phase transition and the telomerase activity.

## Introduction

Dental follicle cells (DFCs) are the precursor cells of periodontal tissues that exhibit stem cell characteristics, such as self-renewal and multilineage differentiation potential. DFCs generally differentiate into cementoblasts, periodontal ligament cells and alveolar bone cells. However, DFCs are multipotent cells–when stimulated by the appropriate signals, they can also differentiate into adipocytes, chondrocytes and neural cells [1], [2]. In addition, *in vitro* culture of DFCs is feasible and involved with minimized ethical considerations, as dental follicles are readily obtained from the impacted wisdom teeth that are routinely removed in orthodontics. Thus, when appropriately stimulated, DFCs might represent a promising cellular resource for periodontal tissue regeneration [3]. However, these cells can easily lose their self-renewal capacity and differentiate into terminal cell types *in vitro* [4], [5]. Moreover, DFCs are difficult to culture and reproduce on a large scale *in vitro*. These characteristics are unfavorable, as a large number of stem cells are required for cell replacement therapy. Therefore, understanding the mechanisms underlying the long-term maintenance of self-renewal capacity and proliferation of these cells *in vitro* represents an important goal in periodontal regeneration research for improving the utility of DFCs.

Notch signaling plays a crucial role in the cell fate decisions of the multipotent precursor cells of metazoans [6]. In mammals, there are four different Notch receptors (Notch1,2,3 and 4) and 5 different Notch ligands (Jagged 1, Jagged 2, Delta-like 1, Delta-like 3, and Delta-like 4). Notch receptors and their ligands are single-pass transmembrane proteins located on the surfaces of adjacent cells. Notch signaling is initiated through the interaction of extracellular ligands with Notch receptors, leading to the sequential cleavage of the Notch extra- and intracellular domains. Once cleaved, the intracellular domain of Notch (ICN) translocates to the nucleus, where it interacts with RBP-Jk (also called CBF1) and activates the transcription of specific target genes, including those of the Hes and Hey family genes. Similarly, the overexpression of ICN, the active form of Notch, activates Notch signaling without ligand binding.

The effects of Notch signaling on individual cells are highly dependent on signal dose and context [7]. Notch signaling is typically associated with cell fate restrictions through the lateral inhibition of cell differentiation; however, this pathway is also widely used in the induction of cell fate interactions [7]. Consistent with a role in cell fate decisions, Notch signaling either promotes or suppresses proliferation, depending on the cellular context [8], [9]. Pathway crosstalk, post-translational modifications, proteolytic processing, endocytosis, membrane trafficking and interactions with the actin cytoskeleton contribute to the diverse effects of Notch signaling [7], [10]. However, the effect of Notch signaling on specific cell types remains largely unstudied.

Telomerase reverse transcriptase (TERT), the catalytic subunit of telomerase, is of vital importance in activating telomerase. High expression of hTERT is often used as a landmark for pluripotency and multipotency state of human embryonic and adult stem cells. Previous studies have shown the expression of TERT and activities of telomerase in DFCs [5], [11], [12], yet their relation to the Notch signaling remains unknown.

Morsczeck et al. originally reported that Notch1 is expressed in cultured human dental follicle cells (HDFCs) [13]. Substantial evidence has shown that Notch1 signaling plays a critical role in the regulation of cell proliferation, differentiation and cell fate decisions in multipotent precursor cells [7]–[9], implicating Notch1 signaling in the regulation of HDFCs growth.

Currently, however, this hypothesis remains unsubstantiated. The purpose of this study was to investigate the role and mechanism(s) underlying Notch1 signaling in the proliferation and self-renewal of HDFCs.

## Materials and Methods

### Ethics Statement

Impacted human third molars were surgically removed during orthodontic surgical procedures from three patients (one 12-year-old boy, one 13-year-old boy and one 14-year-old girl). All the three patients had no systemic and oral infections and diseases except presenting with class III malocclusions. Informed written consents were obtained from the patients and their parents. The study has been approved by the local medical ethics committee and performed in accordance with the regional and international ethics committee guidelines.

### Cell Culture

The HDFCs were cultured as previously described [14], [15]. At passage 4, the HDFCs were subjected to immunocytochemical analysis using antibodies (Table S1) against vimentin, keratin, CD29, CD34, Nestin and Stro-1 according to the method described previously in our lab [15]. Preliminary studies have shown no differences in the morphology and proliferation of HDFCs among different donors, therefore, the HDFCs from the 12-year-old boy were chosen for the studies hereafter.

Both the human erythroleukemic K562 and retroviral packaging 293T cell lines were purchased from a cell bank (Chinese Academy of Sciences). The K562 cells were maintained in RPMI 1640 medium (Gibco) supplemented with 10% FBS (Gibco). The 293T cells were cultured in DMEM (Hyclone) containing 10% FBS at 37°C in a humidified atmosphere containing 95% air and 5% CO_2_.

### Plasmid Construction

The intracellular domain (codon 1770 to 2555) encoding a constitutively active form of Notch1 was amplified by RT-PCR using mRNA extracted from K562 cells. The PCR was performed using forward (5′-ATG TTC CCT GAG GGC TTC AA) and reverse (5′-TTA GTT TTG TGG CTG CAC CTG CT) primers. The DNA fragment was cloned into the pGEM®-T Easy Vector (Promega) and subjected to sequence analysis. The correct DNA fragment was subsequently cloned into pQCXIN (Clontech). A vector containing the enhanced green fluorescent protein gene (pLEGFP-C1; Clontech) was used as a control.

### Retrovirus Preparation and Infection of HDFCs

The packaging 293T cell line was transfected with the retroviral vectors using Lipofectamine2000 (Invitrogen). The 293T cells were treated with 0.6 mg/ml Geneticin (Gibco) at 48 h after transfection. The supernatants from confluent cultures of the Geneticin-resistant producer cells were filtered. After selection with 0.6 mg/ml Geneticin for 2 weeks, the resistant clones were expanded and used to produce viral supernatants. The viral titers were determined through the infection of NIH3T3 cells in the presence of Polybrene (final concentration, 8 µg/ml; Sigma). The titer was greater than 1×10^5^ colony-forming units (cfu)/ml, and no wild-type virus was detected. The HDFCs were seeded into 6-well culture plates at a density of 5×10^5^ cells/well. After culture for 24 h, the cells were incubated with the viral supernatants supplemented with Polybrene (final concentration, 8 µg/ml) at 37°C, 5% CO_2_ for 2–4 h. The cells were washed and cultured in fresh medium overnight. Second and third infections were subsequently performed using the same procedure. The infected HDFCs were selected using 0.2–0.4 mg/ml Geneticin for 2 weeks. The Geneticin-selected HDFCs infected with GFP or ICN1 were designated as HDFC-GFP or HDFC-ICN, respectively. The uninfected parental HDFCs were used as negative controls (HDFC-C).

### Notch1 shRNA Lentiviral Particles Transduction

Notch1 shRNA lentiviral particles (sc-36095-V) and control lentiviral particles expressing a scrambled shRNA (sc-108080) were purchased from Santa Cruz Biotechnology. The HDFCs were seeded into 6-well culture plates at a density of 5×10^5^ cells/well. After culture for 24 h, the cells were transduced with Notch-1 shRNA lentiviral particles and control shRNA lentiviral particles respectively according to the manufacturer's instructions. The successfully tranduced cells were selected by 5 µg/ml Puromycin dihydrochloride (sc-108071) for 3 weeks. The Puromycin-selected HDFCs infected with control shRNA lentiviral particles or Notch1 shRNA lentiviral particles were designated as HDFC-CS or HDFC-NS, respectively.

### Quantitative Real-time RT-PCR

The gene expression levels of Notch1 in five different HDFC groups (HDFC-C, HDFC-GFP, HDFC-ICN, HDFC-CS and HDFC-NS) were assessed by qPCR. The cells were cultured in DMEM containing 10% FBS. At approximately 80% confluence, the cells were starved for an additional 24 h and were subsequently harvested for qPCR. Total RNA was extracted from the cells using an RNeasy Mini Kit (Qiagen) according to the manufacturer’s protocol. One microgram of total RNA from each sample was subjected to first-strand cDNA synthesis using a High Capacity RNA-to-cDNA Master Mix (Applied Biosystems) in a 20-µl-total reaction volume. The reverse transcription reaction was performed at 25°C for 10 min, followed by 48°C for 30 min and 95°C for 5 min. A quantitative PCR reaction was performed using SYBR Green on an ABI PRISM 7700 Sequence Detection System (Applied Biosystems). The primers used are listed in Table S2. The primers were verified through virtual PCR, and the primer concentrations were optimized to avoid primer dimer formation. The thermal profile for the SYBR real-time PCR was 95°C for 10 min, followed by 40 cycles at 95°C for 15 s and 60°C 1 min. A melting curve analysis was performed to verify the specificity of the products. The relative quantification of gene expression was performed using a Comparative CT method according to the manufacturer’s protocol and was normalized to the expression levels of β-actin in each sample.

### Western Blot Analysis

The expression of cleaved Notch1 protein among the five different HDFC groups was determined using western blot analysis. Briefly, the attached cells were rinsed with ice-cold phosphate-buffered saline (PBS), and the cells were scraped on ice into RIPA buffer. The cells were collected in 1.5 ml Eppendorf tubes, lysed on ice for 30 min, and centrifuged at 15,000 rpm for 10 min at 4°C to remove the cellular debris. The protein concentrations were determined using the Bradford assay (Bio-Rad). Equal amounts of proteins were analyzed by 10% SDS-polyacrylamide gel electrophoresis. After electrophoresis, the proteins were electrophoretically transferred onto nitrocellulose membranes (Whatman, Clifton, NJ) using transfer buffer (25 mM Tris, 190 mM glycine, 20% methanol) in a Hoefer TE70XP transfer apparatus (Holliston, MA). The membranes were blocked with skim milk for 60 min and then incubated overnight at 4°C with antigen-specific antibodies for the detection of cleaved Notch1 (#4147, Cell Signaling) and for β-actin (#8457, Cell Signaling). After washing, the membranes were incubated with horseradish peroxidase-conjugated secondary antibodies (#7074, Cell Signaling) for 60 min. The signal intensity of the protein bands was measured by chemiluminescence using a ChemiDoc XRS (Bio-Rad).

### RBP-Jk Luciferase Reporter Assay

RBP-Jk luciferase reporter assays were performed to assess Notch1 signaling activity. The RBP-Jk reporter kit (CCS-014L) was purchased from SABiosciences. The kit contains transfection-ready RBP-Jk reporter construct as well as positive and negative controls. The HDFC-C, HDFC-GFP, HDFC-ICN, HDFC-CS and HDFC-NS cells were seeded into 96-well culture plates at a density of 2×10^4^ cells/well. After culture for 24 h, the cells were transfected with RBP-Jk reporter, negative control and positive control using Lipofectamine 2000™ (Invitrogen) according to the manufacturer's instructions. About 48 h after transfection, luciferase activity was assessed using a Dual-Luciferase reporter assay kit (Promega) according to the manufacturer’s protocol. Luminescence was read using the Veritas Microplate Luminometer (Turner Biosystems). All luciferase activity was normalized with the renilla luciferase activity.

### Cell Cycle Analysis

The cells were seeded in 75 cm^2^ culture flasks. At approximately 80% confluence, the cells were starved for an additional 24 h. Then, single cell suspensions containing at least 5×10^5^ cells were generated and analyzed within 6 h. After washing with PBS, the cells were stained using a DNA-Prep stain kit (Beckman-Coulter). Flow cytometric cell cycle analysis was performed using an ELITE ESP flow cytometer (Beckman-Coulter), and the data were analyzed using Multicycle software (Phoenix Flow Systems).

### Detection of Gene and Protein Expression of Cell Cycle Regulators

The cells were cultured in DMEM containing 10% FBS. At approximately 80% confluence, the cells were starved for an additional 24 h and then harvested. We investigated the gene expression of cell cycle regulators in the five different HDFC groups by qPCR using an identical procedure as described above. The primers used are listed in Table S2.

The protein expression of the cell cycle regulators in the five different HDFC groups was assessed by western blot analysis using an identical procedure to that described above. The antibodies used are listed in Table S3.

### Cell Proliferation Assay

#### Cell number counting

The cells were seeded into 12-well plates at a density of 1×10^4^ cells/well. The DMEM medium containing 10% FBS was changed every 3 days. At each time point of the proliferation assays (0, 1, 3, 5, 7 d), the cells were trypsinized and counted in a hemocytometer using the method of trypan-blue extrusion (Sigma).

#### MTT assay

The cells were seeded into 96-well plates at a density of 4×10^3^ cells/well. At the indicated time points, 100 µl of medium was replaced with an equal volume of fresh medium (DMEM medium containing 10% FBS). Subsequently, 20 µl of MTT stock solution (5 mg/ml; Sigma) was added to each well and incubated for 4 h. The supernatants were removed, and 150 µl of dimethyl sulfoxide was added. After shaking at room temperature for 10 min, the absorbance of each well was measured at 490 nm using a microspectrophotometer (Bio-Tek).

### Detection of Gene Expression of Human Telomerase Reverse Transcriptase (hTERT)

The cells were cultured in DMEM containing 10% FBS. At approximately 80% confluence, the cells were starved for an additional 24 h and then harvested. The gene expression levels of hTERT in the five different HDFC groups were analyzed by qPCR using an identical procedure as described above. The primers used are listed in Table S2.

### Telomerase Activity Assay

The cells were cultured in DMEM containing 10% FBS. At approximately 80% confluence, the cells were starved for an additional 24 h and then harvested for telomerase activity assay. Telomerase activity was measured using the TeloTAGGG Telomerase PCR ELISA kit according to the manufacturer’s instructions (Roche). Briefly, cells were lysed and the protein concentrations were determined using the Bradford assay (Bio-Rad). For each telomerase reaction, 2 µg of proteins were added to the reaction mixture and the reaction was performed at 25°C for 20 min followed by denaturation at 94°C, 5 min, 30 cycles (94°C for 30 s, 50°C for 30 s, and 72°C for 90 s). Final elongation was carried out at 72°C for 10 min. 5 µl PCR amplified products were used for ELISA according to the manufacturer’s instructions. Telomerase activity was expressed as absorbance value measured using a microtiter plate reader (Bio-Rad) at 450 nm with a reference wavelength of 630 nm. The telomerase activity in HDFC-C group was considered as 100% for comparison with the other four groups.

### Statistical Analysis

All of the experiments were replicated at least three times. All of the numerical results were expressed as mean values ± standard deviation (SD). Statistical analyses were performed using the SPSS16.0 software package for Windows. All data were normally distributed. Student’s t test was used for two-group comparisons, and one-way ANOVA test was used for comparisons of 3 or more groups, followed by Tukey’s *post hoc* test. Differences were considered significant when *P*<0.05 (two-tailed).

## Results and Discussion

**Figure 1:**
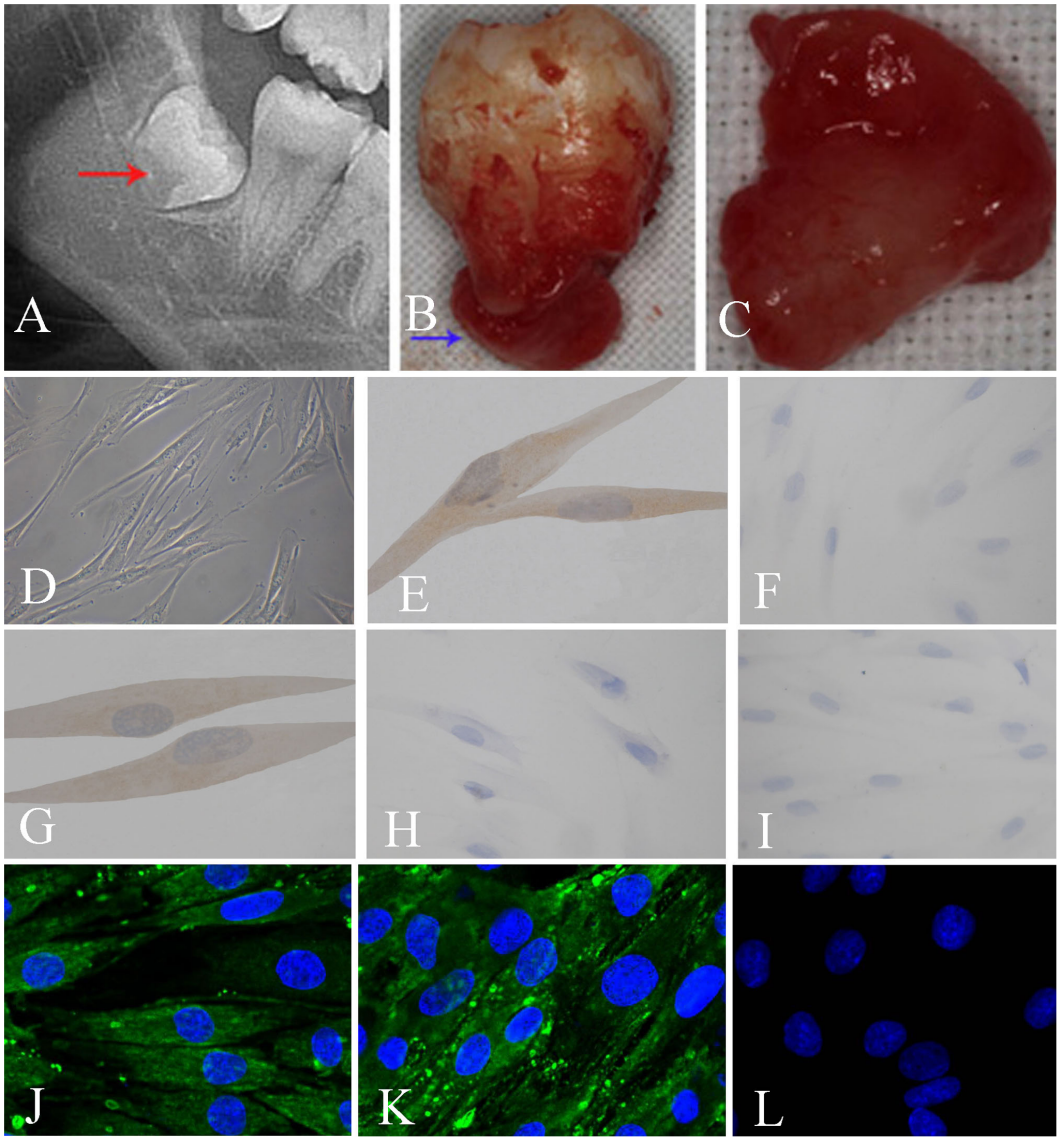
Representative diagrams of processing, culturing and identification of human dental follicle cells (HDFCs). (**A**) unerupted lower third molar and its follicle (arrow) in the OPG radiograph; (**B**) extracted lower third molar surrounding tissues; (**C**) isolated human dental follicle; (**D**) HDFCs in the fourth passage (magnification: 100×); (**E**) positive immunostaining for vimentin in the fourth passage HDFCs (1000×); (**F**) negative immunostaining for keratin in the fourth passage HDFCs (400×); (**G**) positive immunostaining for CD29 in the fourth passage HDFCs (1000×); (**H**) negative immunostaining for CD34 in the fourth passage HDFCs (400×); (**I**) the negative control (400×); (**J**) positive immunostaining for Nestin in the fourth passage HDFCs (800×); (**K**) positive immunostaining for Stro-1 in the fourth passage HDFCs (800×); (**L**) the negative control (800×).

In this study, we investigated the role and mechanism underlying Notch1 signaling in the proliferation and self-renewal of HDFCs. The HDFCs from the three donors were all successfully cultured and exhibited fibroblast-like spindle shapes. In consistent with our previous study [15], the HDFC cell phenotypes were positive for vimentin (a mesenchymal cell marker), CD29 (a mesenchymal stem cell marker), Nestin (a neural stem cell marker) and Stro-1 (a mesenchymal stem cell marker), and negative for keratin (an epithelial cell marker) and CD34 (a hematopoietic stem/progenitor cell marker) (Fig. 1). These results indicated that the cultured cells were mesenchymal cells with stem cell characteristics. The HDFCs from one donor (12-year-old boy) have been chosen for the further investigation.

**Figure 2:**
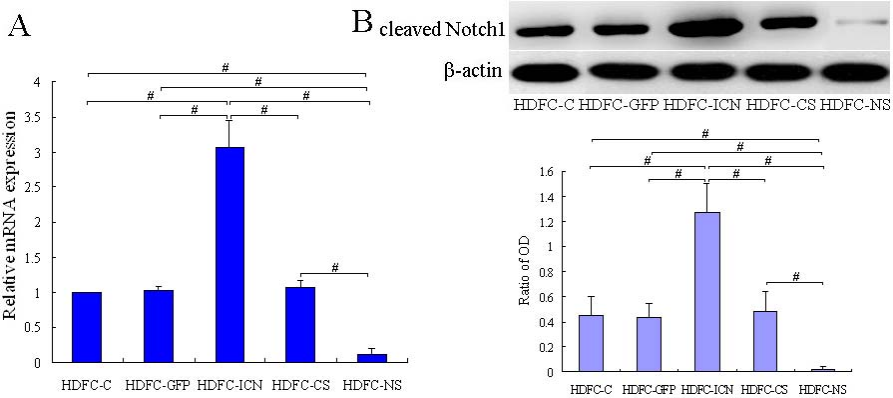
The mRNA and protein expression levels of Notch1 in different HDFC groups. HDFC-C (uninfected parental HDFCs), HDFC-GFP (HDFCs infected with GFP gene), HDFC-ICN (HDFCs infected with the ICN1 gene), HDFC-CS (HDFCs infected with control shRNA lentiviral particles) and HDFC-NS (HDFCs infected with Notch-1 shRNA lentiviral particles) cells were cultured in DMEM containing 10% FBS. At approximately 80% confluence, these cells were starved for an additional 24 h and harvested for qPCR and western blot analyses. (**A**) qPCR analysis of Notch1 transcript levels in different HDFC groups. The data are normalized to β-actin levels and presented as mean values ± SD of three independent experiments. ^#^ *P*<0.01. (**B**) Western blot analysis of cleaved Notch1 protein levels in different HDFC groups. The representative blots show the expression of cleaved Notch1 protein, whereas the bar graph below shows the photodensitometric analysis of the bands of cleaved Notch1 protein, using β-actin as an internal control. The data are presented as mean values ± SD of three independent experiments. ^#^ *P*<0.01.

**Figure 3:**
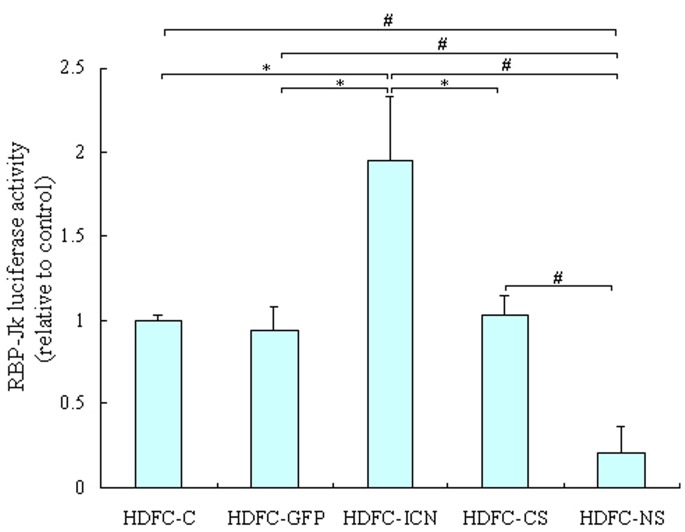
RBP-Jk luciferase reporter activities in different HDFC groups. The HDFC-C, HDFC-GFP, HDFC-ICN, HDFC-CS and HDFC-NS cells were cultured in DMEM containing 10% FBS. At approximately 80% confluence, the cells were starved for an additional 24 h and harvested for RBP-Jk luciferase reporter assay. The data are presented as mean values ± SD of three independent experiments. *P<0.05, ^#^ *P*<0.01.

To analyze the intracellular events induced in the HDFCs by Notch1 regulation, we attempted to reconstitute *ex vivo* systems for the activation (or inhibition)of the Notch1 signaling pathway. A vector containing an exogenous ICN1 gene was constructed and transduced into the HDFCs using a retroviral expression system. The data obtained from the qPCR and western blot analyses indicated that the mRNA expression levels of ICN1 increased (3.06-fold) in the HDFC-ICN cells compared to the HDFC-C or HDFC-GFP cells (Fig. 2A). A 2.82-fold increase in the level of cleaved Notch1 protein was observed in HDFC-ICN cells (Fig. 2B), whereas relatively low levels of cleaved Notch1 protein were expressed in the control (HDFC-GFP and HDFC-C) cells. To knock down Notch1 signaling, the HDFCs were transduced with the lentiviral particles containing the Notch1 shRNA sequences. The mRNA expression level of ICN1 decreased almost 90%, while the level of cleaved Notch1 protein decreased about 95% in the HDFC-NS cells when compared to the control cells (Fig. 2). Furthermore, RBP-Jk luciferase reporter assays were performed to assess the Notch1 activity in different HDFC groups. The data showed that the Notch1 activity increased (1.95-fold) in the HDFC-ICN cells, while decreased almost 80% in the HDFC-NS cells when compared to the control cells (Fig. 3). These results confirmed that successful establishment of the Notch1-overexpressing HDFCs (HDFC-ICN) and Notch1-silencing HDFCs (HDFC-NS).

**Figure 4:**
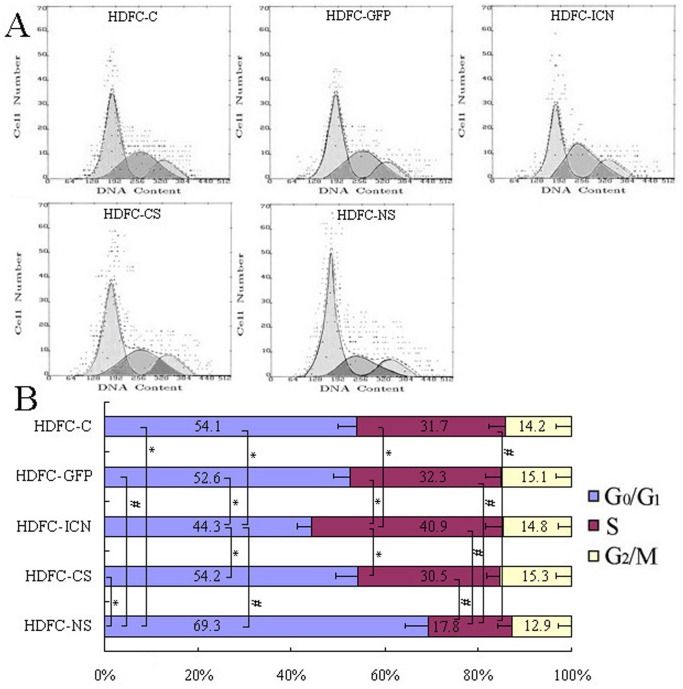
The effect of Notch1 regulation on the cell cycle dynamics of the HDFCs. The HDFC-C, HDFC-GFP, HDFC-ICN, HDFC-CS and HDFC-NS cells were cultured in DMEM containing 10% FBS. At approximately 80% confluence, the cells were starved for an additional 24 h and harvested for cell cycle analysis by flow cytometry. (**A**) The plots show the representative cell cycle distributions of three independent experiments. (**B**) The bar graph represents the average results of the percentage of cells in G0/G1, S, and G2/M phases. The data are presented as mean values ± SD of three independent experiments. *P<0.05, ^#^ *P*<0.01.

The cell cycle has been proposed to serve as a “gatekeeper” for self-renewal and is closely linked with cell proliferation. Notch1 signaling is implicated in cell cycle control, in addition to other diverse cellular behaviors and mechanisms [16], [17]. Therefore, we investigated how Notch1 regulation affects the cell cycle progression of HDFCs. The flow cytometric analysis revealed that the stable expression of exogenous ICN1 significantly reduced the number of cells in the G0/G1 phase and increased the number of cells in the S phase compared with control cells, whereas the cells in the G2/M phase remained virtually unchanged (Fig. 4). Thus, constitutively active Notch1 appears to reduce the number of the G1 phase cells and accelerates the S phase transition in HDFCs. In contrast, Notch1 silencing results in a significant increase in the number of cells within the G0/G1 phase and a significant decrease in the number of cells in the S phase compared with the control cells (Fig. 4). Although some studies have shown that Notch1 activation induces G0/G1 phase cell cycle arrest [18], the results obtained from this study are consistent with published data indicating that Notch1 activation (or inhibition) promotes (or delays) the G1/S transition [19]–[21]. These results also demonstrate that the effects of Notch signaling are cell-type-specific and context-dependent. G1 phase is a particularly important part of the cell cycle and determines whether a cell remains in the proliferative state or executes other cell fate decisions. Therefore, a shortened G1 phase and an accelerated S-phase transition induced by Notch1 activation may diminish the ability of HDFCs to differentiate, promoting their self-renewal capacity and proliferation.

**Figure 5:**
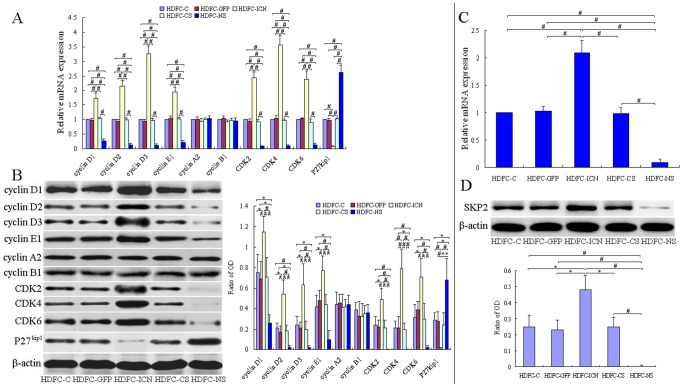
The effect of Notch1 regulation on the expression of cell cycle regulators and SKP2 in different HDFC groups. The HDFC-C, HDFC-GFP, HDFC-ICN, HDFC-CS and HDFC-NS cells were cultured in DMEM containing 10% FBS. At approximately 80% confluence, the cells were starved for an additional 24 h and harvested for qPCR and western blot analyses. (**A**) qPCR analysis of the transcript levels of different cell cycle regulators in the different HDFC groups. The data are normalized to β-actin levels and presented as mean values ± SD of three independent experiments. ^#^ *P*<0.01. (**B**) Western blot analysis of the protein levels of different cell cycle regulators in the different HDFC groups. The representative blots show the protein expression levels of the different cell cycle regulators, and the bar graph represents the results from the photodensitometric analysis of the bands of the different cell cycle regulators, using β-actin as an internal control. The data are presented as mean values ± SD of three independent experiments. *P<0.05, ^#^ *P*<0.01. (**C**) qPCR analysis of SKP2 transcript levels in the different HDFC groups. The data are normalized to β-actin levels and presented as mean values ± SD of three independent experiments. ^#^ *P*<0.01. (**D**) Western blot analysis of the SKP2 protein levels in the different HDFC groups. The representative blots show the expression of the SKP2 protein, and the bar graph below represents the results from the photodensitometric analysis of the bands of SKP2 protein, using β-actin as an internal control. The data are presented as mean values ± SD of three independent experiments. *P<0.05, ^#^ *P*<0.01.

Cell cycle progression is regulated through a complex network involving cyclins, cyclin-dependent kinases (CDKs), and cyclin-dependent kinase inhibitors (CKIs). To elucidate the mechanisms underlying the cell cycle regulation of Notch1-overexpressing (or Notch1-silencing) HDFCs, we examined changes in the expression of different cell cycle regulators during this process. The D-type cyclins (cyclin D1, D2, and D3) are induced in cells entering the G1 phase of the cell cycle. Following mitogenic stimulation, D-type cyclins are synthesized, which bind to and activate CDKs such as CDK4 and CDK6. The activation of CDKs in the G1 phase regulates the phosphorylation and inactivation of the retinoblastoma protein (Rb), as well as the derepression of E2F transcription factors, driving the cell into the S phase [22]. We found that Notch1 activation upregulates cyclin D1, cyclin D2, cyclin D3, CDK4 and CDK6 (Fig. 5). The increased expression of these cell cycle regulators shortened G1 and accelerated the G1/S-phase transition. While in the Notch1 silencing group, the opposite results were obtained (Fig. 5). These data are consistent with previous reports regarding the contribution of Notch1 signaling to the G1/S-phase transition via the upregulated expression of cyclin D1, cyclin D3, CDK4 and CDK6 [20], [23]. Here, we provide evidence that inhibition of Notch1 signaling downregulated the expression of the above mentioned factors, and in addition cyclin D2 may also be involved in this process.

Cyclin E1 and CDK2 are two activators of the late G1/S phase cell cycle checkpoint, and the activities of these two activators are also required for the G1/S phase transition. Our results showed that the expression of cyclin E1 and CDK2 was upregulated through the activation of Notch1 (Fig. 5), suggesting that these activators might also represent the factors that limit the G1/S-phase transition in HDFCs. These findings are consistent with recent data showing that porcine satellite cell proliferation is associated with significant changes in the expression of cell cycle-related genes including cyclin E1 [8]. Moreover, the current study further showed that the inhibition of Notch1 signaling downregulated the expression of cyclin E1 and CDK2 thus impact the cell cycle in the opposite way. Previous studies have demonstrated that CDK2 activation results from the degradation of the CDK inhibitor protein p27^ kip1^, which is triggered by the Notch1-induced expression of SKP2, a component of the ubiquitin ligase complex, which targets proteins for proteosomal degradation [19], [24]. Consistent with the literature, our data showed a significant decrease in the p27^Kip1^ expression in correlation with a significant increase in the SKP2 expression after Notch1 activation, while the opposite changes were observed in the Notch1 inhibition group (Fig. 5).

Cyclin A2 is required for both G1/S and G2/M transitions and plays a role in stimulating DNA synthesis. Unexpectedly, our results indicated that Notch1 activation or inhibition elicits no effect on the gene and protein expression levels of cyclin A2 (Fig. 5). Previous studies of mouse embryonic stem cells demonstrated that the activation of Notch signaling promotes cell proliferation through the upregulated expression of cyclin D1 but not of cyclin A [19], suggesting that cyclin A might not be responsible for the G1/S-phase transition.

Cyclin B1 is required for progression through the G2/M phase. The results showed that the expression levels of cyclin B1 remained unchanged in Notch1-overexpressing or Notch1-silencing HDFCs (Fig. 5). These results are consistent with our flow cytometric analyses, which show that changes of Notch1 activation elicit no significant effect on the G2/M-phase transition.

**Figure 6:**
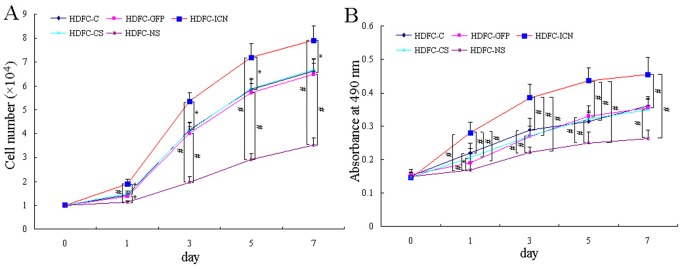
The effect of Notch1 regulation on proliferation of the HDFCs. (**A**) The HDFC-C, HDFC-GFP, HDFC-ICN, HDFC-CS and HDFC-NS cells were seeded into 12-well plates and harvested at the indicated time points for cell number counting. The data are presented as mean values ± SD of three independent experiments. *P<0.05, ^#^ *P*<0.01. (**B**) The HDFC-C, HDFC-GFP, HDFC-ICN, HDFC-CS and HDFC-NS cells were seeded into 96-well plates and harvested at the indicated time points for MTT assay. The data are presented as mean values ± SD of three independent experiments. *P<0.05, ^#^ *P*<0.01.

Moreover, we examined the growth-stimulating effect of Notch1 signaling on cultured HDFCs using cell number counting and MTT assay. As shown in Fig. 6, the proliferation of the HDFCs increased in each group in a time-dependent manner. However, the HDFC-ICN group exhibited higher proliferation while the HDFC-NS group showed less proliferation than the corresponding control group (*P*<0.05), except at the baseline time point (day 0). The results demonstrated that the stable expression of constitutively active Notch1 significantly stimulates the growth of the HDFCs, while inhibition of Notch1 signaling suppresses the growth of the HDFCs *in vitro*. Based on the strong correlation between the altered cell cycle dynamics and the Notch1-overexpressing/Notch1-silencing in HDFCs, it is reasonable to conclude that Notch1 signaling regulates the proliferation of human dental follicle cells by modulating the G1/S phase transition.

**Figure 7:**
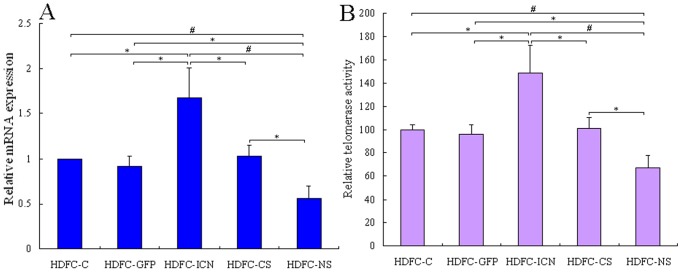
hTERT mRNA expression levels and telomerase activities in different HDFC groups. The HDFC-C, HDFC-GFP, HDFC-ICN, HDFC-CS and HDFC-NS cells were cultured in DMEM containing 10% FBS. At approximately 80% confluence, the cells were starved for an additional 24 h and harvested for qPCR and telomerase activity assays. (**A**) qPCR analysis of hTERT transcript levels in different HDFC groups. The data are normalized to β-actin levels and presented as mean values ± SD of three independent experiments. *P<0.05, ^#^ *P*<0.01. (**B**) Relative telomerase activity by TRAP-ELISA assays in different HDFC groups. The data are presented as mean values ± SD of three independent experiments and the telomerase activity in HDFC-C cells was considered as 100% for comparison with the other four groups. *P<0.05, ^#^ *P*<0.01.

To determine whether Notch1 signaling could maintain HDFCs self-renewal capacity and undifferentiated state, we compared the gene expression levels of hTERT and telomerase activities in different HDFC groups. The data clearly showed that the gene expression levels of hTERT increased by 70% in the HDFC-ICN cells while decreased about 45% in the HDFC-NS cells when compared to the control cells (*P*<0.05). Correspondingly, the telomerase activity increased almost 50% in the HDFC-ICN cells and decreased about 35% in the HDFC-NS cells when compared to the control cells (*P*<0.05, Fig. 7). These observations are pivotal because upregulation of hTERT and the high level of telomerase activity were usually the features of cells that divide rapidly, including both embryonic stem cells and adult stem cells [25]. Upregulation of hTERT elongates the telomeres of stem cells which consequently prolongs the lifespan of the stem cells. Elongating the telomeres in the cells can lead to the indefinite division. Therefore, it is responsible for the self-renewal properties of stem cells. Furthermore, previous studies have shown that both telomerase activity and the hTERT gene are either undetectable or expressed at an extremely low level in most human differentiated cells [26], [27]. Hence, the results indicated that Notch1 activation helped to maintain HDFCs self-renewal capacity and repress HDFCs differentiation by upregulating hTERT expression as such increase the telomerase activity. This may provide an explanation to our previous findings that Notch1 activation can also inhibit the osteoblastic differentiation of the bone marrow stromal cells [28].

Based on the findings of the current study, Notch1 signaling promotes the proliferation and maintains the self-renewal capacity of HDFCs. However, these results require further detailed investigation. The HDFCs comprise heterogeneous cellular subpopulations with different proliferation rates, morphologies, and differentiation potentials [29]. We do not know whether all of the follicular cells or only a select population (e.g., the progenitors for cementoblasts and alveolar osteoblasts) respond to the regulation of Notch1 signaling. If all of the heterogeneous HDFC subpopulations are responsive to Notch1 regulation, whether Notch1 signaling uniformly affects the proliferation of the HDFC subpopulations is unclear. Furthermore, we examined the role of Notch1 overexpression or silencing in HDFCs proliferation *in vitro*; whether the conclusions drawn here can be applied *in vivo* remains unknown. Thus, animal studies are needed to confirm the role and mechanisms underlying Notch1 signaling in HDFCs proliferation.

### Conclusions

The current study clearly showed the proliferation and self-renewal of HDFCs can be enhanced via constitutive activation of Notch1 and suppressed by Notch1 inhibition *in vitro*. The stimulation of HDFCs growth is associated with the increased expression of cyclin D1, cyclin D2, cyclin D3, cyclin E1, CDK2, CDK4, CDK6, and SKP2 and the reduced expression of p27^ kip1^. Changes in the expression of the cell cycle regulators shortened the G1 phase and accelerated the S-phase transition. Meanwhile, Notch1 activation upregulated the gene expression of hTERT and increased the telomerase activity. A shortened G1 phase in combination with upregulated hTERT expression can diminish the ability of HDFCs to differentiate, thus promote their proliferation and self-renewal capability. Our findings deepen the understanding towards the molecular mechanisms of the regulation of HDFCs proliferation and self-renewal through Notch1 signaling, which would provide cues and clues to improve future application of HDFCs in periodontal tissue regeneration.

## Supporting Information

Table S1 **Primary and secondary antibodies used for immunocytochemistry assay.**
(DOC)

Table S2 **Primer sequences used for real-time PCR.**
(DOC)

Table S3 **Primary and secondary antibodies used for western blot analysis.**
(DOC)

## References

[1] Morsczeck C, Götz W, Schierholz J, Zeilhofer F, Kühn U, et al (2005) Isolation of precursor cells (PCs) from human dental follicle of wisdom teeth. Matrix Biol 24: 155–165. 15890265 10.1016/j.matbio.2004.12.004

[2] Völlner F, Ernst W, Driemel O, Morsczeck C (2009) A two-step strategy for neuronal differentiation in vitro of human dental follicle cells. Differentiation 77: 433–441. 19394129 10.1016/j.diff.2009.03.002

[3] Honda MJ, Imaizumi M, Tsuchiya S, Morsczeck C (2010) Dental follicle stem cells and tissue engineering. J Oral Sci 52: 541–552. 21206155 10.2334/josnusd.52.541

[4] Yokoi T, Saito M, Kiyono T, Iseki S, Kosaka K, et al (2007) Establishment of immortalized dental follicle cells for generating periodontal ligament in vivo. Cell Tissue Res 327: 301–311. 17013589 10.1007/s00441-006-0257-6

[5] Zhou J, Liu T, Zheng H, Song JL, Deng F (2012) Immortalization of the SD rats' dental follicle cell with simian virus 40 large tumor antigen gene. Zhonghua Kou Qiang Yi Xue Za Zhi 47: 631–636. 23302389 10.3760/cma.j.issn.1002-0098.2012.10.013

[6] Fiúza UM, Arias AM (2007) Cell and molecular biology of Notch. J Endocrinol 194: 459–474. 17761886 10.1677/JOE-07-0242

[7] Schwanbeck R, Martini S, Bernoth K, Just U (2011) The Notch signaling pathway: molecular basis of cell context dependency. Eur J Cell Biol 90: 572–581. 21126799 10.1016/j.ejcb.2010.10.004

[8] Qin L, Xu J, Wu Z, Zhang Z, Li J, et al (2013) Notch1-mediated signaling regulates proliferation of porcine satellite cells (PSCs). Cell Signal 25: 561–569. 23160004 10.1016/j.cellsig.2012.11.003

[9] Schroeder T, Just U (2000) mNotch1 signaling reduces proliferation of myeloid progenitor cells by altering cell-cycle kinetics. Exp Hematol 28: 1206–1213. 11063868 10.1016/s0301-472x(00)00534-8

[10] D'Souza B, Miyamoto A, Weinmaster G (2008) The many facets of Notch ligands. Oncogene 27: 5148–5167. 18758484 10.1038/onc.2008.229 PMC2791526

[11] d'Aquino R, Tirino V, Desiderio V, Studer M, De Angelis GC, et al (2011) Human neural crest-derived postnatal cells exhibit remarkable embryonic attributes either in vitro or in vivo. Eur Cell Mater 21: 304–316. 21432784 10.22203/ecm.v021a23

[12] Jeon BG, Kang EJ, Kumar BM, Maeng GH, Ock SA, et al (2011) Comparative analysis of telomere length, telomerase and reverse transcriptase activity in human dental stem cells. Cell Transplant 20: 1693–1705. 21396170 10.3727/096368911X565001

[13] Morsczeck C, Moehl C, Götz W, Heredia A, Schäffer TE, et al (2005) In vitro differentiation of human dental follicle cells with dexamethasone and insulin. Cell Biol Int 29: 567–575. 15951208 10.1016/j.cellbi.2005.03.020

[14] Chen XP, Qian H, Wu JJ, Ma XW, Gu ZX, et al (2007) Expression of vascular endothelial growth factor in cultured human dental follicle cells and its biological roles. Acta Pharmacol Sin 28: 985–993. 17588334 10.1111/j.1745-7254.2007.00586.x

[15] Chen XP, Duan YZ, Qian H, Jin ZL (2012) Effect of protein kinase C (PKC) and protein kinase A (PKA) signaling pathways on the expression of VEGF in cultured human dental follicle cells. Chinese Journal of Cell Biology 34: 447–453.

[16] Artavanis-Tsakonas S, Rand MD, Lake RJ (1999) Notch signaling: cell fate control and signal integration in development. Science 284: 770–776. 10221902 10.1126/science.284.5415.770

[17] Noseda M, Karsan A (2006) Notch and minichromosome maintenance (MCM) proteins: integration of two ancestral pathways in cell cycle control. Cell Cycle 5: 2704–2709. 17172856 10.4161/cc.5.23.3515

[18] Duan L, Yao J, Wu X, Fan M (2006) Growth suppression induced by Notch1 activation involves Wnt-beta-catenin down-regulation in human tongue carcinoma cells. Biol Cell 98: 479–490. 16608439 10.1042/BC20060020

[19] Sarmento LM, Huang H, Limon A, Gordon W, Fernandes J, et al (2005) Notch1 modulates timing of G1-S progression by inducing SKP2 transcription and p27 Kip1 degradation. J Exp Med 202: 157–168. 15998794 10.1084/jem.20050559 PMC2212905

[20] Joshi I, Minter LM, Telfer J, Demarest RM, Capobianco AJ, et al (2009) Notch signaling mediates G1/S cell-cycle progression in T cells via cyclin D3 and its dependent kinases. Blood 113: 1689–1698. 19001083 10.1182/blood-2008-03-147967 PMC2647664

[21] Borghese L, Dolezalova D, Opitz T, Haupt S, Leinhaas A, et al (2010) Inhibition of notch signaling in human embryonic stem cell-derived neural stem cells delays G1/S phase transition and accelerates neuronal differentiation in vitro and in vivo. Stem Cells 28: 955–964. 20235098 10.1002/stem.408

[22] Vermeulen K, Van Bockstaele DR, Berneman ZN (2003) The cell cycle: a review of regulation, deregulation and therapeutic targets in cancer. Cell Prolif 36: 131–149. 12814430 10.1046/j.1365-2184.2003.00266.x PMC6496723

[23] Das D, Lanner F, Main H, Andersson ER, Bergmann O, et al (2010) Notch induces cyclin-D1-dependent proliferation during a specific temporal window of neural differentiation in ES cells. Dev Biol 348: 153–166. 20887720 10.1016/j.ydbio.2010.09.018

[24] Dohda T, Maljukova A, Liu L, Heyman M, Grandér D, et al (2007) Notch signaling induces SKP2 expression and promotes reduction of p27Kip1 in T-cell acute lymphoblastic leukemia cell lines. Exp Cell Res 313: 3141–3152. 17560996 10.1016/j.yexcr.2007.04.027

[25] Flores I, Benetti R, Blasco MA (2006) Telomerase regulation and stem cell behaviour. Curr Opin Cell Biol 18: 254–260. 16617011 10.1016/j.ceb.2006.03.003

[26] Wright WE, Piatyszek MA, Rainey WE, Byrd W, Shay JW (1996) Telomerase activity in human germline and embryonic tissues and cells. Dev Genet 18: 173–179. 8934879 10.1002/(SICI)1520-6408(1996)18:2<173::AID-DVG10>3.0.CO;2-3

[27] Wang S, Zhao Y, Hu C, Zhu J (2009) Differential repression of human and mouse TERT genes during cell differentiation. Nucleic Acids Res 37: 2618–2629. 19270068 10.1093/nar/gkp125 PMC2677880

[28] Xing Q, Ye Q, Fan M, Zhou Y, Xu Q, et al (2010) Porphyromonas gingivalis lipopolysaccharide inhibits the osteoblastic differentiation of preosteoblasts by activating Notch1 signaling. J Cell Physiol 225: 106–114. 20648628 10.1002/jcp.22201

[29] Luan X, Ito Y, Dangaria S, Diekwisch TG (2006) Dental follicle progenitor cell heterogeneity in the developing mouse periodontium. Stem Cells Dev 15: 595–608. 16978062 10.1089/scd.2006.15.595 PMC2738600

