# Supplementary material for: Notch1 Signaling Regulates the Proliferation and Self-Renewal of Human Dental Follicle Cells by Modulating the G1/S Phase Transition and Telomerase Activity
Source: PLoS One. 2013 Jul 29;8(7):e69967. doi: 10.1371/journal.pone.0069967 (PMC3726724; doi:10.1371/journal.pone.0069967)
Supplement: Table S1 — Primary and secondary antibodies used for immunocytochemistry assay. (DOC) [file pone.0069967.s001.doc]

**Table S1: Primary and secondary antibodies used for immunocytochemistry assay**

| Primary antibody | Catalog number | Company |
| --- | --- | --- |
| vimentin | IR630 | Dako |
| keratin | Z0622 | Dako |
| CD29 | sc-9970 | Santa Cruz |
| CD34 | sc-19621 | Santa Cruz |
| Nestin | sc-23927 | Santa Cruz |
| Stro-1 | sc-47733 | Santa Cruz |
|  |  |  |
| Secondary antibody |  |  |
| HRP-conjugated goat anti-mouse IgG | A5278 | Sigma |
| HRP-conjugated goat anti-rabbit IgG | [A9169](http://www.sigmaaldrich.com/catalog/product/sigma/a9169?lang=zh&region=CN) | Sigma |
| FITC-conjugated goat anti-mouse IgG | F9006 | Sigma |
