# Supplementary material for: Notch1 Signaling Regulates the Proliferation and Self-Renewal of Human Dental Follicle Cells by Modulating the G1/S Phase Transition and Telomerase Activity
Source: PLoS One. 2013 Jul 29;8(7):e69967. doi: 10.1371/journal.pone.0069967 (PMC3726724; doi:10.1371/journal.pone.0069967)
Supplement: Table S2 — Primer sequences used for real-time PCR. (DOC) [file pone.0069967.s002.doc]

**Table S2: Primer sequences used for real-time PCR**

| Gene | Sequence (5' to 3') |
| --- | --- |
| Notch1 | Forward: CACTGTGGGCGGGTCC |
|  | Reverse: GTTGTATTGGTTCGGCACCAT |
| cyclin D1 | Forward: TGCCCTCTGTGCCACAGATG |
|  | Reverse: TCTGGAGAGGAAGCGTGTGA |
| cyclin D2 | Forward: TGCTCTGTGTGCCACCGACTT |
|  | Reverse: CAGCTCAGTCAGGGCATCACAA |
| cyclin D3 | Forward: TTTGCCATGTACCCGCCATCCA |
|  | Reverse: CCCGCAGGCAGTCCACTTCA |
| cyclin E1 | Forward: CGGCTCGCTCCAGGAA |
|  | Reverse: TCATCTGGATCCTGCAAAAAAA |
| cyclin A2 | Forward: ACAGTAAACAGCCTGCGTTC |
|  | Reverse: AAGAGGGACCAATGGTTTTC |
| cyclin B1 | Forward: GGCCAAAATGCCTATGAAGA |
|  | Reverse: GATGTTTCCATTGGGCTTG |
| CDK2 | Forward: TGGATGCCTCTGCTCTCACTG |
|  | Reverse: GAGGACCCGATGAGAATGGC |
| CDK4 | Forward: GAAACTCTGAAGCCGACCAG |
|  | Reverse: GGCAGAGATTCGCTTGTGT |
| CDK6 | Forward: GCACAGTGTCACGAACAGA |
|  | Reverse: CCTCGGAGAAGCTGAAACA |
| P27^kip1^ | Forward: CCCTAGAGGGCAAGTACGAGT |
|  | Reverse: AGTAGAACTCGGGCAAGCTG |
| SKP2 | Forward: GCTGCTAAAGGTCTCTGGTGT |
|  | Reverse: AGGCTTAGATTC TGCAACTTG |
| hTERT | Forward: AGGGGCAAGTCCTACGTCCAGT |
|  | Reverse: CACCAACAAGAAATCATCCACC |
| β-actin | Forward: GGCATCCTCACCCTGAAGTA |
|  | Reverse: GGGGTGTTGAAGGTCTCAAA |
