# Supplementary material for: Notch1 Signaling Regulates the Proliferation and Self-Renewal of Human Dental Follicle Cells by Modulating the G1/S Phase Transition and Telomerase Activity
Source: PLoS One. 2013 Jul 29;8(7):e69967. doi: 10.1371/journal.pone.0069967 (PMC3726724; doi:10.1371/journal.pone.0069967)
Supplement: Table S3 — Primary and secondary antibodies used for western blot analysis. (DOC) [file pone.0069967.s003.doc]

**Table S3: Primary and secondary antibodies** used for western blot analysis

| Primary antibody | Catalog number | Company |
| --- | --- | --- |
| cyclin D1 | #2978 | Cell Signaling |
| cyclin D2 | ab94685 | Abcam |
| cyclin D3 | sc-6283 | Santa Cruz |
| cyclin E1 | ab101324 | Abcam |
| cyclin A2 | #4656 | Cell Signaling |
| cyclin B1 | ab72 | Abcam |
| CDK2 | ab6433 | Abcam |
| CDK4 | ab108357 | Abcam |
| CDK6 | ab151247 | Abcam |
| P27^kip1^ | ab137736 | Abcam |
| SKP2 | ab19877 | Abcam |
| β-actin | #8457 | Cell Signaling |
|  |  |  |
| Secondary antibody |  |  |
| HRP-conjugated anti-rabbit | #7074 | Cell Signaling |
| HRP-conjugated anti-mouse | #7076 | Cell Signaling |
